# How to manage food allergy in restaurants, cafeterias and fast food outlets?

**DOI:** 10.1186/2045-7022-1-S1-S59

**Published:** 2011-08-12

**Authors:** Frans Timmermans

**Affiliations:** 1 European Anaphylaxis Taskforce - Nederlands Anafylaxis Netwerk, Dordrecht, Netherlands

## Background

The stress of raising a child with food allergies affects each family differently, depending, in large part, on how the physician initially presents the information and the family's coping style. Based on how the information is presented they can leave the doctor's office terrified and unsure whether they will be able to prevent the next reaction, or concerned but confident that they can keep their child safe. We educate patients to be informed and to communicate and are encouraged to "live a normal life while taking precautions".

## Methods

Food businesses were asked different aspects, by questionnaire and interview, on how they dealt with food allergic guest.

## Results

95% of the respondents (n=115) indicated that they would provide a safe meal, but only 26% has had a food allergen training. 55% of the respondents believed that heat would destroy the allergen en 46% believed that a small amount of allergen would do no harm. About half of the respondents thought that a buffet would be safe if it was kept clean and some 21% thought that removing the allergen from a prepared meal (like nut topping) would make the meal safe to eat. 85% recognized peanut, tree nuts, milk and egg as main allergens and 18% indicated that they had a procedure to provide allergy safe meals. None of the respondents had an action plan in case of an allergic emergency other than to call 112. 78% thought that regulation on food safety and food hygiene did not include food allergens.

## Conclusion

The encouraging part is restaurant and catering businesses in the Netherlands are willing to provide allergy safe meals to their allergic guests but would like to be informed in advance.

The lack of knowledge on food allergy and allergens indicated the necessity for more education and training. Food allergic guests have to present themselves, but still need to be extra careful. A solution could be the proposal for a new EU regulation on the provision of food information to final consumers, as this will be a big step forward in safeguarding the food allergic individual.

